# Ultrashort Echo Time Quantitative Susceptibility Mapping (UTE-QSM) of Highly Concentrated Magnetic Nanoparticles: A Comparison Study about Different Sampling Strategies

**DOI:** 10.3390/molecules24061143

**Published:** 2019-03-22

**Authors:** Xing Lu, Hyungseok Jang, Yajun Ma, Saeed Jerban, Eric Y. Chang, Jiang Du

**Affiliations:** 1 Department of Radiology, University of California San Diego, San Diego, CA 92103, USA; lvxingvir@gmail.com (X.L.); h4jang@ucsd.edu (H.J.); yam013@ucsd.edu (Y.M.); sjerban@ucsd.edu (S.J.); ericchangmd@gmail.com (E.Y.C.); 2 Institute of Electrical Engineering, Chinese Academy of Science, Beijing 100190, China; 3 Radiology Service, Veterans Affairs San Diego Healthcare System, San Diego, CA 92037, USA

**Keywords:** magnetic resonance imaging, magnetic nanoparticles, quantitative susceptibility mapping, ultrashort echo imaging

## Abstract

The ability to accurately and non-invasively quantify highly concentrated magnetic nanoparticles (MNPs) is desirable for many emerging applications. Ultrashort echo time quantitative susceptibility mapping (UTE-QSM) has demonstrated the capability to detect high iron concentrations. In this study, we aimed to investigate the effect of different sampling trajectories on the accuracy of quantification based on MNPs acquired through UTE-QSM. A phantom with six different MNP concentrations was prepared for UTE-QSM study with different UTE sampling trajectories, including radial acquisition, continuous single point imaging (CSPI), and Cones with four different gradient stretching factors of 1.0, 1.2, 1.4, and 1.6. No significant differences were found in QSM values derived from the different UTE sampling strategies, suggesting that the UTE-QSM technique could be accelerated with extended Cones sampling.

## 1. Introduction

In the past ten years, magnetic nanoparticles (MNPs) have been used in many medical branches, such as cell labeling, drug delivery, magnetic resonance imaging (MRI), and magnetic hyperthermia for cancer therapy [1,2]. Magnetic thermal therapy for cancer treatment kills cancer cells by increasing the local temperature. Currently, there are two kinds of heating treatments: hyperthermia to stimulate the immune response for non-specific immunotherapy (41–46 °C) [3] and thermo-ablation (46–56 °C) to initiate cell necrosis/coagulation/carbonization for tumor destruction [4]. With this approach, tumors can be destroyed with minimal harm to healthy tissues, thus reducing the negative side effects associated with the delivery of drugs. In this way of cancer therapy, MNPs are injected directly into the tumor; then, an external alternating magnetic field with a specific frequency between 50 kHz to 1.2 MHz is applied to heat the MNPs [5]. Accurate knowledge of the distribution of the MNPs within the body is important for treatment planning [6]. The ability to accurately and non-invasively quantify highly concentrated MNPs is closely related to the rapidly growing area of magnetic biosensing [7,8,9]. Magnetic resonance imaging (MRI), which is free from ionizing radiation and allows for rich contrast mechanisms, is naturally suitable for imaging of the MNPs in vivo. 

Quantitative susceptibility mapping (QSM) has recently emerged as a promising technique in MRI to quantitatively measure magnetic susceptibility using magnetic resonance phase images, which can provide clinically important information such as iron deposition, calcification, demyelination, and oxygenation [10,11,12,13,14,15]. QSM typically utilizes gradient recalled echo (GRE) imaging to obtain the phase images. Therefore, QSM may allow for the detection of the MNPs distribution in the human body with more specific physical meanings [16]. However, the MNPs concentration required for efficient hyperthermia is generally greater than 18 mM (1 mg/mL) of iron [17]. Currently, conventional clinical GRE sequences are unable to quantify the concentration of MNPs in the therapeutic range because the MRI signal of iron above 9 mM—which is the upper limit of regular MRI techniques in assessing highly concentrated MNPs [17]—is dominated by noise, even at the shortest possible echo times due to its ultrashort T2* value.

Advanced techniques with much reduced echo time (TE), including ultrashort echo time (UTE) imaging [18,19], zero echo time (ZTE) imaging [20], hybrid encoding [21,22], and sweep imaging with Fourier transformation (SWIFT) [23], make it possible to accurately quantify highly concentrated MNPs with MRI [17,24,25,26]. Our previous preliminary study showed that UTE-QSM with three-dimensional (3D) Cones trajectories could increase the detection range of iron concentrations (up to 22 mM). Shorter first echo time and shorter echo spacing were shown to be key factors contributing to increased detection range [27]. Following that study, we successfully implemented 3D continuous single point imaging (CSPI)-based UTE-QSM for the estimation of high iron concentrations [28]. Besides Cones and CSPI, conventional radial or projection reconstruction (PR)-based UTE-QSM has also been used for the evaluation of ultrashort T2* tissues, such as cortical bone [29]. 

In this study, we aimed to investigate the effect of different UTE sampling strategies on the QSM-based evaluation of MNPs concentration. UTE images acquired at different TEs were used together with a morphology-enabled dipole inversion (MEDI) algorithm to estimate QSM values [30]. A phantom with different iron concentrations ranging from 2 to 22 mM was built to systematically compare three different UTE sampling strategies including PR, CSPI, and Cones. Moreover, different gradient stretching factors in Cones imaging were also compared to investigate the effect of acceleration on QSM quantification accuracy. 

## 2. Materials and Methods

### 2.1. 3D Ultrashort Echo Time Imaging Schemes

**Figure 1:**
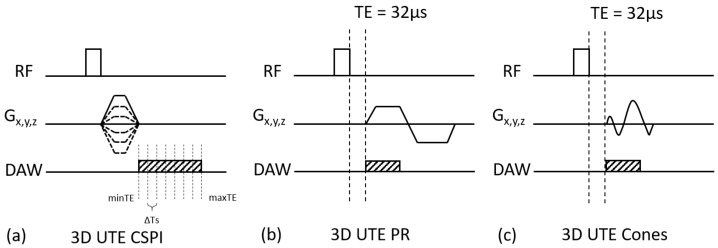
Three different sequences for the comparative ultrashort echo time quantitative susceptibility mapping (UTE-QSM) studies: (**a**) the 3D UTE continuous single point imaging (CSPI) sequence with a minimum echo time (minTE) of 528 μs, (**b**) the 3D UTE projection reconstruction (PR) (radial) sequence with a minimum TE of 32 μs, (**c**) the 3D UTE Cones sequence with a minimum TE of 32 μs. (RF means Radio Frequency pulse, DAW means Data Acquisition Window).

In this study, three different UTE imaging schemes were compared in QSM estimation: 3D UTE-CSPI, 3D UTE-PR, and 3D UTE-Cones sequences. Figure 1a shows a pulse sequence diagram (PSD) for 3D UTE-CSPI, where gradients were switched on, rapidly ramped up with maximum slew rate after RF excitation, ramped down, and switched off once the desired resolution (or spatial frequency) was achieved. After switching off the gradients, data were continuously acquired at a fixed k-space location, where myriad k-space was acquired with extremely high temporal resolution (e.g., echo spacing of 2 μs) in a single scan. CSPI is based on pure phase encoding, where the readout duration is nearly zero, which allows one to capture a true snapshot of the phase in transverse magnetization. For more time-efficient encoding, CSPI can be performed with acceleration techniques (e.g., parallel imaging or compressed sensing). In this study, generalized autocalibrating partially parallel acquisitions (GRAPPA) was utilized to further accelerate CSPI [31]. Figure 1b shows a PSD for 3D UTE-PR imaging, which utilized a straight radial sampling trajectory. In the PR imaging, trapezoidal gradients were applied to achieve fast encoding of the quickly decaying UTE signal. Figure 1c shows a PSD for 3D UTE-Cones imaging, where a short rectangular pulse excitation was used, followed by data readout with efficient Cones spiral trajectory with a minimal nominal TE of 32 µs (a minimal TE of 8 µs could be achieved with the addition of a fast transmit/receive switch) [32,33,34,35]. The 3D k-space was divided into multiple cones, with twisted spiral trajectories along each cone. The trajectory could be made more or less twisted (i.e., longer or shorter spiral arms), which was associated with a relative stretching factor (SF). Typically, more gradient stretching meant a longer readout duration, which reduced the scan time at the cost of shorter T2* blurring or a chemical shift artifact (intra-voxel signal interference). 

### 2.2. Iron Phantom Design

**Figure 2:**
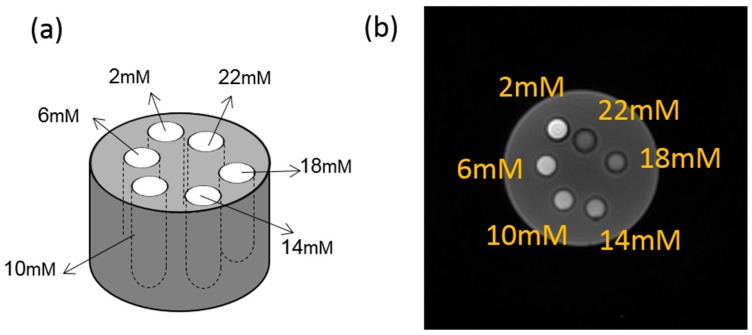
(**a**) Demonstration of the phantom design. (**b**) Typical magnitude image of the first echo with the UTE-Cones sequence at TE = 32 μs.

An iron phantom was prepared with six tubes, each filled with 2 mL of six different concentrations of Feridex I.V.^®^ solution (Berlex Laboratories, Wayne, NJ, USA). Feridex I.V.^®^ (ferumoxides injectable solution) is a sterile aqueous colloid of superparamagnetic iron oxide associated with dextran for intravenous administration as a magnetic resonance imaging contrast media. Chemically, ferumoxide is a non-stoichiometric magnetite of average formula FeO_1.44_. Each milliliter of Feridex I.V.^®^ contains 11.2 mg of iron. By diluting the original Feridex I.V.^®^ solution in purified water, the concentrations of each tube by weight were 2, 6, 10, 14, 18, and 22 mM. As shown in Figure 2, the tubes were placed in a cylinder container (10 cm in diameter, 30 cm in height) and filled with agarose gel (0.9% by weight). 

### 2.3. Imaging Experiment

The three different UTE imaging schemes (i.e., UTE-CSPI, UTE-PR, and UTE-Cones) were used to scan the iron phantom on a 3T clinical MR system (MR750, GE Healthcare, Waukesha, WI, USA) using a 16-channel receive-only wrap coil (NeoCoil, Pewaukee, WI, USA), with the longitudinal direction of the tubes placed parallel to the B_0_ field. The imaging parameters are summarized in Table 1. 

For parallel imaging in UTE-CSPI, the following imaging parameters were used: acceleration factor (AF) = 2 × 2 × 1, matrix size for auto-calibration = 17 × 17 × 100. To further investigate the effects of readout-gradient stretching, the UTE-Cones imaging was repeated with four different SFs: 1.0 (default, AF = 2.6 over UTE-PR sampling), 1.2 (AF = 3.5), 1.4 (AF = 4.1), and 1.6 (AF = 5.0). The stretching factor was defined as the ratio between the sampling window using the stretched spiral sampling and the sampling window using the PR trajectory, while AF was defined as the ratio of total scan time of PR sequence to each Cones sequence. The higher SF required a smaller number of spokes to cover the 3D k-space and, therefore, required a shorter scan time. The other parameters were kept the same. 

### 2.4. Image Reconstruction

UTE-Cones and UTE-PR images were reconstructed using offline reconstruction codes based on non-uniform fast Fourier transform (NuFFT) [36]. After the k-space data in each channel were reconstructed, the images at individual channels were combined to form a complex image [37]. CSPI images were reconstructed utilizing 3D GRAPPA reconstruction using a kernel size of 5 × 5 × 7 pixels. To enhance signal-to-noise ratio (SNR) in CSPI, consecutive k-spaces within a small TE window (±16 μs) were temporally averaged after GRAPPA reconstruction as in [28].

### 2.5. QSM Analysis

Each 3D UTE-Cones acquisition was reconstructed into both magnitude and phase images using a re-gridding algorithm. Nominal TEs were used for QSM calculation. The MEDI QSM reconstruction algorithm was applied offline with the same complex matrix for measuring the susceptibility of each iron phantom [30,38]. For all datasets, the regularization parameter λ and radius for the spherical mean value operator were kept as 500 and 5, respectively, for calculating magnetic susceptibility χ. User-defined regions of interest (ROIs) with fixed diameters of 1 cm were used to cover each tube. All data processing was performed using Matlab 2017b (Mathworks Inc., Natick, MA, USA).

## 3. Results

### 3.1. Comparison between UTE Techniques

**Figure 3:**
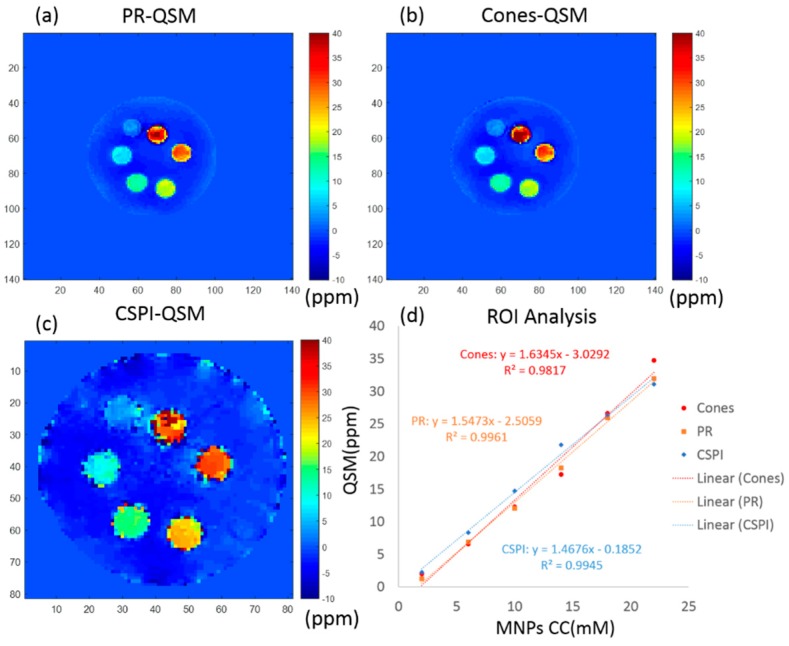
UTE-QSM) results of an iron phantom obtained with three different sequences: (**a**) 3D UTE-PR (PRQSM), (**b**) 3D UTE-Cones (Cones-QSM), and (**c**) 3D UTE CSPI (CSPI-QSM). Region of interest (ROI) analyses of different vials showed an excellent linear relationship between UTE-QSM and iron concentration for all three sequences (**d**) MNPs: magnetic nanoparticles.

Figure 3 shows the QSM results of all three UTE imaging techniques (CSPI, PR, and Cones) with the same scale of susceptibility (ppm). Note that the UTE-CSPI sequence has a smaller sampling field of view (FOV) than UTE-PR and UTE-Cones sequences, so the iron phantom in Figure 3c looks bigger than in Figure 3a,b. The resolution was kept the same for all three images. With the UTE-Cones (SF = 1.0), the mean and standard deviation of the estimated susceptibility in each tube was 2.0 ± 1.3, 6.6 ± 2.2, 12.3 ± 2.6, 17.3 ± 3.1, 26.6 ± 4.2, and 34.7 ± 9.0 ppm, respectively. The estimated mean susceptibility showed high linearity (R^2^ = 0.9817) with the fitted equation for susceptibility, $\chi_{\mathrm{Cones}}$ = 1.6345[Fe] − 3.0292 (ppm). For the UTE-PR sequence, the mean and standard deviation of the estimated susceptibility in each tube was 1.3 ± 2.1, 6.9 ± 2.0, 12.1 ± 3.1, 18.3 ± 1.4, 25.9 ± 4.6, and 32.0 ± 8.3 ppm, respectively. The estimated mean susceptibility showed high linearity (R^2^ = 0.9961) with the fitted equation for susceptibility, $\chi_{\mathrm{PR}}$ = 1.5473[Fe] − 2.5059 (ppm). For the UTE-CSPI sequence, the mean and standard deviation of the estimated susceptibility in each tube was 2.3 ± 1.3, 8.3 ± 3.1, 14.7 ± 3.6, 21.8 ± 5.0, 26.4 ± 12.4, and 31.1 ± 21.3 ppm, respectively. The estimated mean susceptibility showed high linearity (R^2^ = 0.9945) with the fitted equation for susceptibility, $\chi_{\mathrm{CSPI}}$ = 1.4676[Fe] − 0.1852 (ppm).

### 3.2. Comparison between Different Stretching Factors in UTE-Cones

**Figure 4:**
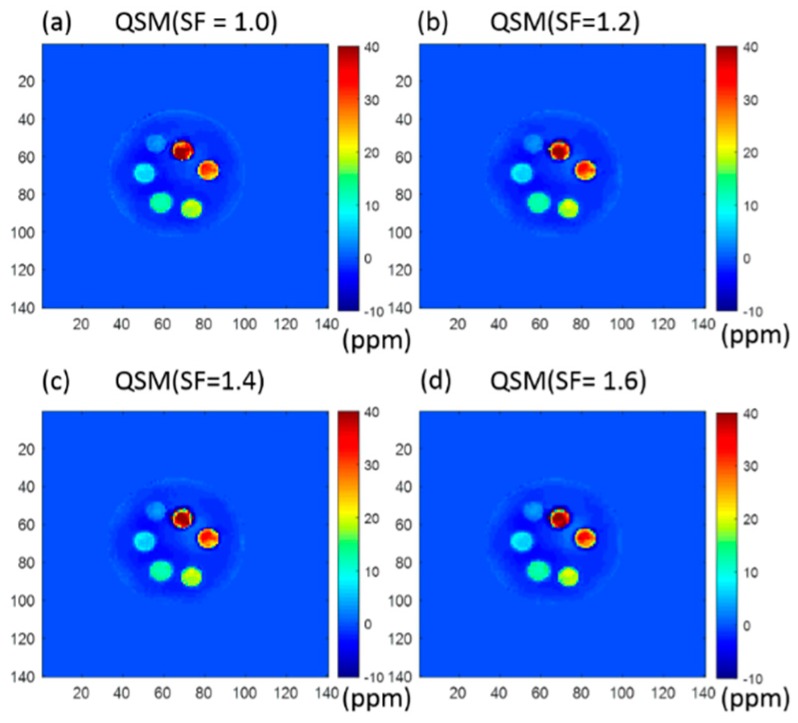

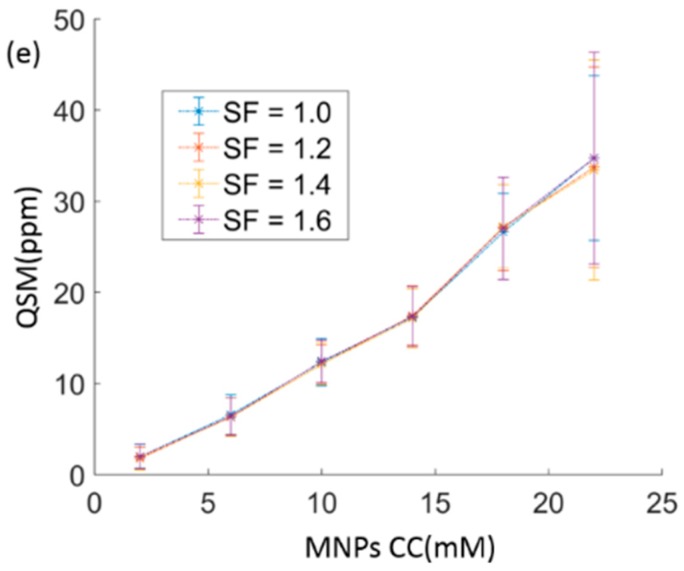
Cones-QSM with four different stretching factors of (**a**) 1.0, (**b**) 1.2, (**c**) 1.4, and (**d**) 1.6, respectively. The ROI analysis of different iron concentrations is shown in (**e**). Similar UTE-QSM values were achieved with different stretching factors, except for the tube with the highest iron concentration of 22 mM, which showed lower QSM values and greater standard deviations with higher stretching factors.

Figure 4 shows typical QSM results of Cones sequences with four different stretching factors (SF = 1.0, 1.2, 1.4, and 1.6).

For UTE-Cones with an SF of 1.2, the means and standard deviations of the estimated susceptibility in each tube were 1.8 ± 1.2, 6.4 ± 2.0, 12.1 ± 2.1, 17.5 ± 3.3, 27.1 ± 4.7, and 33.7 ± 11.0 ppm, respectively. For UTE-Cones with an SF of 1.4, the means and standard deviations of the estimated susceptibility in each tube were 1.9 ± 1.4, 6.3 ± 2.1, 12.1 ± 2.3, 17.1 ± 3.3, 27.2 ± 4.6, and 33.4 ± 12.1 ppm, respectively. For UTE-Cones with an SF of 1.6, the means and standard deviations of the estimated susceptibility in each tube were 1.0 ± 1.4, 6.4 ± 2.1, 12.4 ± 2.3, 17.3 ± 3.3, 27.0 ± 5.6, and 34.7 ± 11.6 ppm, respectively. As can be seen, QSM estimates of different SFs showed very consistent results for most of the tubes between UTE-Cones with different SFs, except for the 22 mM tube, where slightly higher QSM values were shown with SFs of 1.0 and 1.6 than with the other two SFs.

## 4. Discussion

The performance of QSM combined with various UTE imaging techniques was investigated for fast and accurate evaluation of highly concentrated MNPs. The relatively long scan time in UTE-QSM is a significant barrier for widespread clinical adoption. Accelerated UTE-QSM may facilitate future clinical studies associated with high iron concentrations, such as magnetic hyperthermia therapy, hemophilia, etc. In this study, we compared the performance of different UTE-QSM techniques based on 3D PR, CSPI, and Cones with different stretching factors ranging from 1.0 to 1.6. The phantom studies suggested that the Cones sequence had similar accuracy to PR and CSPI sequences in the UTE-QSM assessment of MNPs concentration. Furthermore, a higher stretching factor of up to 1.6, which corresponds to an acceleration factor of 5, did not significantly affect the QSM estimation. To the best of our knowledge, this is the first study comparing different UTE sampling strategies for the UTE-QSM assessment of high MNPs concentrations. The fact that Cones-QSM with a high stretching factor allows fast and accurate estimation of highly concentrated MNPs is of significant clinical importance, and may play a role in advancing the translational study of iron mapping.

The CSPI sequence is believed to provide the most accurate phase information, owing to its near-zero readout duration [16]. However, the scan time imposed by 3D phase encoding is a crucial issue for clinical applications. The 3D Cones sequence is based on center-out spiral readout trajectories, where the k-space data are encoded on the surface of multiple cones, allowing more efficient encoding than other UTE imaging techniques. When using similar imaging parameters, as shown in Table 1, the Cones sequence required a shorter scan time than both CSPI and PR sequences. The phantom experiments show that the estimated QSM values were almost the same with three different UTE imaging techniques. This indicates that the Cones sequence allowed accurate evaluation of MNPs concentrations from 2 to 22 mM within a much shorter scan time, which is beneficial for clinical applications. The concentration range covered the therapeutic range threshold of 18 mM. Higher concentrations of MNPs could also be evaluated with UTE-Cones with shorter echo spacing time, according to our previous study [27]. 

Another advantage of UTE-Cones imaging is that each spiral arm can be stretched on the conical surface to more efficiently cover the surface of each cone in 3D k-space. Therefore, a much-reduced number of spiral arms (or readouts) is required to cover one cone, which can further reduce the total scan time. Moreover, the stretched spiral arms in UTE-Cones allow a more uniform sampling density, which may improve SNR efficiency [36,39]. In our study, as seen in Table 1, changing SF from 1 to 1.6 reduced the scan time from 156 s to 92 s. However, a higher SF will introduce increased short T2* blurriness and chemical shift artifacts due to a longer readout duration. Moreover, the effective TE will deviate more from the prescribed TE with a higher SF due to the stretched readout. In the iron phantom experiment, an SF of up to 1.6 did not significantly affect QSM results for the tubes with concentrations from 2 to 18 mM. For the tube with the highest iron concentration of 22 mM, the estimated susceptibility appeared slightly changed with the increased SFs. The instability is presumably caused by increased short T2* blurring, the increased deviation in effective TEs from the prescribed TEs, and increased off-resonance artifacts. Because of these factors, we did not test SF higher than 1.6, which works for low iron concentrations but fails for high iron concentrations.

In this study, the quantitative evaluation of MNPs concentration was based on MRI methodology. Besides MRI, there are many other methodologies that have the ability to detect highly concentrated MNPs, such as magnetic particle imaging (MPI), ultrasound (US), magnetoimpedance, inductive technique, etc. MPI is based on the non-linear magnetization response of the super-magnetic property of the MNPs, and is a promising way to obtain the distribution of MNPs. However, state-of-the-art MPI has limited resolution (~500 μm) and small FOV (<100 mm × 100mm) due to the requirement of a high gradient field, which is not yet applicable for in vivo human studies [40,41]. US is one of the most used biomedical imaging techniques in clinical practice. Recent reports suggest that the signal of magneto-motive ultrasound (MMUS) is proportional to the concentration of the MNPs. However, US has limitations in its resolution, contrast, and penetration depth [42]. Magnetic field detection methods with magnetic induction sensors, such as superconducting quantum interference device (SQUID)-based sensors, giant magneto-impedance (GMI) sensors, and giant magneto-resistance (GMR) sensors, etc., have very high sensitivity (as high as 10^−10^ Oe) for detecting ferromagnetic biomaterials [43,44]. However, these methods are mainly used for detection of a signal from a local area and are not yet applicable for imaging. 

There are several limitations of this study. First, the highest concentration of MNPs in this study was 22 mM, which is lower than the 37.5 mM concentration used in a study of UTE T2* or T1 measurement [25], and the 57.5 mM concentration used in a study of SWIFT [17]. Second, MNPs of different concentrations were suspended homogeneously in our phantom; however, MNPs would be expected to accumulate or aggregate within biological tissues, causing nonhomogeneous susceptibility values in vivo. Third, our study is limited by the absence of in vivo results. However, there are currently no clinical MRI techniques available for the quantitative imaging of highly concentrated MNPs. It is crucial that we first develop novel UTE-QSM techniques for the evaluation of highly concentrated MNPs, such as the Feridex solutions included in this study. Synthetic polymer gels and natural biopolymers, which better mimic biological tissues, will be used for future studies before translating the developed techniques for in vivo imaging [45,46]. Fourth, the chemical shift effect was not considered in this study. UTE-QSM together with the iterative decomposition of water and fat with echo asymmetry and least squares estimation (IDEAL) techniques may help resolve potential issues. As this study focused on the measurable MNPs concentrations with various UTE-QSM sequences, it was unlikely that the chemical shift effect influenced the results of our phantom studies. Fifth, only MEDI-based QSM processing was considered in this study. UTE acquisitions combined with other QSM processing algorithms, such as improved sparse linear equation and least-squares (iLSQR), will be investigated in future studies [47]. 

## 5. Conclusions

In this study we investigated the feasibility of quantitative susceptibility mapping combined with various ultrashort echo time sequences for the fast and accurate estimation of highly concentrated MNPs. Different UTE sampling strategies, including 3D PR, CSPI, and Cones with four different stretching factors were compared, and the results showed no significant difference in QSM values for MNPs up to 22 mM. The Cones-QSM sequence with a stretching factor up to 1.6 can be used for the fast and accurate mapping of high iron concentrations. 

## Figures and Tables

**Table 1:** Scan parameters of three types of ultrashort echo time (UTE) sequences and the Cones sequence with four different stretching factors (SFs) of 1.0, 1.2, 1.4, and 1.6.

3D UTE Sequences	TEs ^#^ (ms)	TR ^##^ (ms)	Resolution (mm)	Matrix	Scan Time (mins)	Bandwidth (kHz)
CSPI	0.528, 0.56, 0.592, 0.624, 0.656, 0.688	7	1 × 1 × 1	80 × 80 × 100	10′56″	±250
PR	0.032, 0.132, 0.232, 0.332	7	1 × 1 × 1	140 × 140 × 100	5′38″ × 4	±83.33
Cones (SF = 1)	0.032, 0.132, 0.232, 0.332	7	1 × 1 × 1	140 × 140 × 100	2′36″ × 4	±83.33
Cones (SF = 1.2)	0.032, 0.132, 0.232, 0.332	7	1 × 1 × 1	140 × 140 × 100	2′03″ × 4	±83.33
Cones (SF = 1.4)	0.032, 0.132, 0.232, 0.332	7	1 × 1 × 1	140 × 140 × 100	1′48″ × 4	±83.33
Cones (SF = 1.6)	0.032, 0.132, 0.232, 0.332	7	1 × 1 × 1	140 × 140 × 100	1′32″ × 4	±83.33

^#^ TEs is Echo Times; ^##^> TR is Repetition Time.
